# DNA sequences alignment in multi-GPUs: acceleration and energy payoff

**DOI:** 10.1186/s12859-018-2389-6

**Published:** 2018-11-20

**Authors:** Jesús Pérez-Serrano, Edans Sandes, Alba Cristina Magalhaes Alves de Melo, Manuel Ujaldón

**Affiliations:** 1 Computer Architecture Department, University of Malaga, Malaga, Spain; 2 Computer Science Department, University of Brasilia, Brasilia, Brazil

**Keywords:** GPGPU, CUDA, DNA sequences alignment, HPC, Power efficiency

## Abstract

**Background:**

We present a performance per watt analysis of CUDAlign 4.0, a parallel strategy to obtain the optimal pairwise alignment of huge DNA sequences in multi-GPU platforms using the exact Smith-Waterman method.

**Results:**

Our study includes acceleration factors, performance, scalability, power efficiency and energy costs. We also quantify the influence of the contents of the compared sequences, identify potential scenarios for energy savings on speculative executions, and calculate performance and energy usage differences among distinct GPU generations and models. For a sequence alignment on chromosome-wide scale (around 2 Petacells), we are able to reduce execution times from 9.5 h on a Kepler GPU to just 2.5 h on a Pascal counterpart, with energy costs cut by 60%.

**Conclusions:**

We find GPUs to be an order of magnitude ahead in performance per watt compared to Xeon Phis. Finally, versus typical low-power devices like FPGAs, GPUs keep similar GFLOPS/w ratios in 2017 on a five times faster execution.

## Background

The rapid evolution of sequencing techniques has produced myriads of data in recent Genome Projects. In this context, data is generated in such a high rate that the traditional analyses tools are not able to cope with them, leading to a dilemma usually called *data deluge*. Consequently, the focus of Genome Projects has moved from data production to data analysis and the central challenge nowadays is how to analyze such a huge amount of data in a rapid and accurate way. In order to face this challenge, biology and computer science joined forces, exploring solutions that demand sophisticated algorithms and powerful computing devices. As a result, refined solutions have been devised to support several biological subdomains such as protein structure prediction and docking [1, 2], sequence comparison [3] and evolutionary biology [4], among others.

High performance computing platforms are composed of several computing cores and can accelerate several algorithms from many research domains, producing results in reduced time. Modern GPUs (graphics processing units) have thousands of cores and have proved to be excellent platforms to run parallel applications that exhibit regular data dependencies. They have been with us for a decade as typical accelerators, and have recently found alternatives in Xeon Phi processors as many-core platforms for HPC and FPGAs as low-power devices. CUDA (Compute Unified Device Architecture) [5] and OpenCL [6] provide valuable support for data and compute intensive applications to exploit the GPU’s powerful engine, making it possible to attain several TFLOPS (Trillions Floating Point Operations per Second) and high speed bandwidth. GPUs have been used as successful platforms for large-scale bioinformatics and many researchers have investigated the behaviour of these applications on GPUs and suggested improvements [7–9].

This work extends the study to energy consumption, an issue of growing interest in the High Performance Computing (HPC) community. GPU-based supercomputers have been for several years in the top500 list (www.top500.org) of the most powerful supercomputers and have recently conquered the green500 (www.green500.org) supercomputer list. In fact, energy consumption sometimes represents more than 20% of the budget in Data Centers, and costs have exceeded 5 billion dollars per year over the last decade only in the US [10]. Many estimations state that energy costs will greatly increase in the near future unless power optimizations are applied in all system levels, from the operating system to the application.

This paper is an extended version of [11]. Our work focuses on pairwise biological sequence alignment, aiming to obtain the degree of similarity between the sequences. Within this topic, there are two basic approaches: (a) global alignment, which aligns the entire length of the sequences and is used for similar sequences in content and size; and (b) local alignment, where high similarity sections within the sequences are highlighted. Needleman-Wunsch (NW) [12] proposed an algorithm for global sequence alignment based on dynamic programming (DP), and Smith-Waterman (SW) [13] adapted the NW algorithm to the local alignment case. Both algorithms have high computational requirements, so researchers either use heuristic methods as in the well-known BLAST tools [14], or rely on high performance computing solutions to reduce the execution time. We have chosen GPUs to explore the latter.

The rest of this paper is organized as follows. “Biological sequence comparison” section describes the problem of comparing two DNA sequences. “Related work” section completes this section with some related work. “CUDAlign implementation on GPUs” section summarizes our previous work on GPUs. “Experimental setup” and “Monitoring energy” sections introduce our infrastructure for measuring the experimental numbers, which are later analyzed in “Results” section. Finally, “Discussion and conclusions” section draws conclusions of this work.

## Methods

## Biological sequence comparison

Biological sequences are composed of an ordered sequence of residues, which can be nucleotides (DNA/RNA sequences) or amino acids (protein sequences) [15]. These sequences are treated as strings composed of elements of the alphabets *Σ*={*A*,*T*,*G*,*C*}, *Σ*={*A*,*U*,*G*,*C*} and *Σ*={*A*,*C*,*D*,*E*,*F*,*G*,*H*,*I*,*K*,*L*,*M*,*N*,*P*,*Q*,*R*,*S*,*T*,*V*,*W*,*Y*}, respectively. Protein and RNA sequences are rather small and their sizes range from hundreds to tens of thousands of residues (amino acids and nucleotide bases, respectively). On the other hand, DNA sequences can be very long, often composed of Millions of Base Pairs (MBP).

### Similarity score and alignment

To compare two sequences, we need to find the best alignment between them, that is, how characters match when you overlap them [16]. In an alignment, spaces (gaps) can be inserted in arbitrary locations along the sequences so that they end up with the same size.

**Fig. 1:**
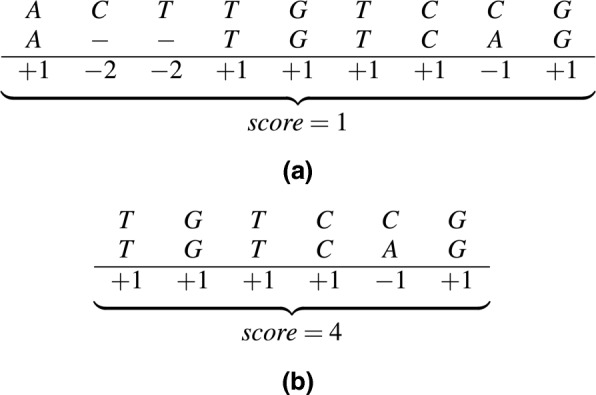
(**a**) Global and (**b**) local alignments and scores. Values for matches, mismatches and gaps are +1, -1 and -2, respectively

In order to measure the quality of a DNA sequence alignment a score is calculated, considering three cases: (a) matches (*ma*), if the characters of both sequences at the same column are identical; (b) mismatches (*mi*), if the characters in the same column are distinct and (c) gaps (gap), if one of the characters in the same column is a space. The score is the sum of all values assigned to the columns and a high score points to high similarity sequences. Figure 1a and b illustrate global and local alignments between two DNA sequences (*S*_0_=ACTTGTCCG and *S*_1_=ATGTCAG).

In Fig. 1, a single value is assigned for matches and mismatches (+1 and −1 in the example) regardless of the parts involved. This works well with nucleotides (DNA or RNA sequences) but not for proteins. During evolution, some combinations are more likely to occur than others, so higher scores are assigned to these combinations [17]. Therefore, the alignment of protein sequences employ scoring matrices, such as PAM (Percent Accepted Mutations) and BLOSUM (Blocks Substitution Matrix), which associate values for matches/mismatches that correspond to the likelihood of a particular combination [15]. PAM matrices have scores that are calculated by analyzing the frequencies in which a given amino acid is substituted by another amino acid during evolution. BLOSUM scoring matrices are created by evaluating evolutionary rates of a region of inside a protein (block) rather than considering the entire protein.

### Exact algorithms: obtaining the optimal alignment

#### Algorithm NW for global alignment

The algorithm proposed by Needleman and Wunsh (NW) [12] is an exact method based on dynamic programming to obtain the optimal global alignment between two sequences in quadratic time and space. In fact, the algorithm originally proposed by NW had cubic time complexity [16], but later on, its complexity was reduced to quadratic, which is the version we describe in this paper. The algorithm is divided in two phases: create the DP matrix and obtain the optimal global alignment.

##### Phase 1 - calculate the DP matrix

In the first phase, the input sequences are *S*_0_ and *S*_1_, with |*S*_0_|=*m* and |*S*_1_|=*n*, where |*S*_*i*_| is the length of sequence *S*_*i*_. For sequences *S*_0_ and *S*_1_, there are *m*+1 and *n*+1 possible prefixes, respectively, including the empty sequence. In order to represent the *n-th* character of a sequence *S*_*i*_, the notation is *S*_*i*_[*n*]. Fianlly, we use *S*_*i*_[1..*n*] to characterize a prefix with *n* characters, from the beginning of the sequence.

In the initialization step, the first row and column are set to −*Gx*, where *x* is the size of the non-empty subsequence and *G* is the gap penalty. This represents the cost of aligning a non-empty subsequence with an empty one. In other words, the first row and column of the DP matrix are initialized with values *H*_0,*j*_=−*Gj* and *H*_*i*,0_=−*Gi*. Additionally, *H*_0,0_=0. The remaining elements of *H* are calculated with the recurrence relation Eq. 1. The optimal score is the value contained in cell *H*_*m*,*n*_.

1 $$ H_{i,j}=\max \left\{\begin{array}{l} H_{i-1,j-1}+p(i,j) \\ H_{i,j-1}-G \\ H_{i-1,j}-G \\ \end{array}\right.  $$

In Eq. 1, if DNA or RNA sequences are compared, *p*(*i*,*j*) is usually the match value (*ma*) if *S*_0_[*i*]=*S*_1_[*j*] or the mismatch penalty (−*mi*), otherwise. If protein sequences are compared, *p*(*i*,*j*) is given by a scoring matrix (e.g. PAM or BLOSUM).

**Fig. 2:**
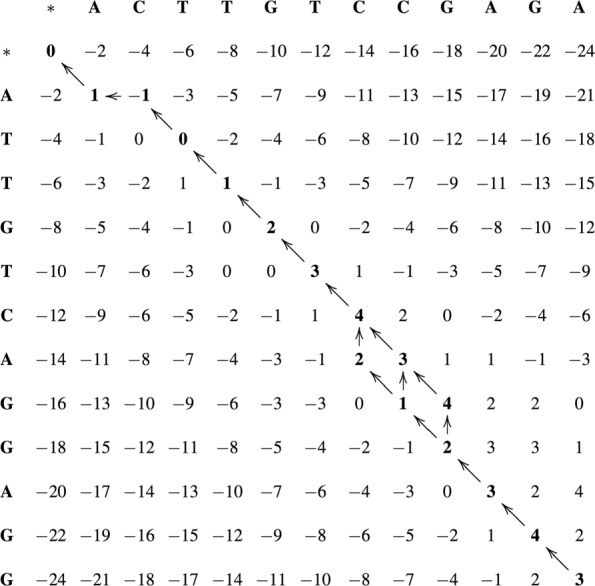
DP matrix for global alignment between sequences *S*_0_ and *S*_1_

Figure 2 presents the DP matrix between sequences *S*_0_ = ATTGTCAGGAGG and *S*_1_ = ACTTGTCCGAGA. The arrows indicate the cell from where the value was obtained, according to Eq. 1. Cells with multiple arrows indicate that the same maximum value was produced by more than one cell (*H*_*i*−1,*j*−1_, *H*_*i*,*j*−1_, *H*_*i*−1,*j*_).

##### Phase 2 - obtain the optimal global alignment

Phase 2 starts from the position that contains the optimal score (*H*_*m*,*n*_) and follows the arrows until cell *H*_0,0_ is reached. A left arrow in *H*_*i*,*j*_ (Fig. 2) produces the alignment of *S*_0_[*i*] with a gap in *S*_1_. An up arrow aligns *S*_0_[*j*] with a gap in *S*_1_. Finally, a diagonal arrow indicates that *S*_0_[*i*] is aligned with *S*_1_[*j*].

Note that many optimal global alignments may exist, since many arrows may be present in the same cell *H*_*i*,*j*_. Usually, the implementations restrict to one the optimal alignment, giving preference to a given type of arrow (diagonal, up, left).

#### Algorithm SW for local alignment

Local alignment must be employed when the goal is to obtain the similarity between regions inside the sequences. Smith and Waterman (SW) [13] proposed an exact algorithm for local alignment 1985 and, since then, this algorithm is widely used. Like NW (“Algorithm NW for global alignment” section), SW is also based on dynamic programming with quadratic time and space complexity. However, there are three basic differences between the algorithms NW and SW, concerning the calculation of the DP matrix (“Phase 1 - calculate the DP matrix” section) and the alignment retrieval.

In the initialization step, all elements of the first row and column are set to zero in SW. This is done because gaps should not receive any penalty at the beginning of the alignment.

The second difference is the recurrence relation itself since since negative values are not allowed in SW, as shown in Eq. 2.

2 $$ H_{i,j}=\max \left\{\begin{array}{l} H_{i-1,j-1}+p(i,j) \\ H_{i,j-1}-G \\ H_{i-1,j}-G \\ 0 \\ \end{array}\right.  $$

In the second phase (*obtain the optimal local alignment*), the algorithm starts from the cell which has the highest value in the DP matrix, following the arrows until a zero-valued cell is reached.

**Fig. 3:**
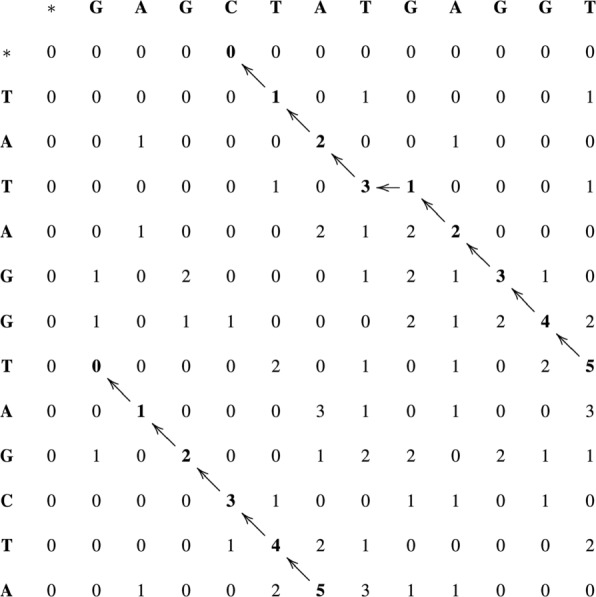
Similarity matrix for local alignment between sequences *S*_0_ and *S*_1_

Figure 3 presents the similarity matrix to obtain local alignments between sequences *S*_0_= TATAGGTAGCTA and *S*_1_= GAGCTATGAGGT. Note that, in this example, even though there are no multiple arrows leaving a single cell, two optimal alignments can be obtained, both of them with score 5. Most of the implementations of SW will only retrieve one of those optimal alignments.

#### Affine-gap

Algorithms NW (“Algorithm NW for global alignment” section) and SW (“Algorithm SW for local alignment” section) use the linear gap model, where each gap is assigned the same penalty. To produce more biologically relevant results, Gotoh [18] proposed an algorithm that implements the affine-gap model for the global alignment case. This model takes into account the observation that, in nature, gaps tend to occur together [17]. In this case, a higher penalty is assigned to open a gap (*G*_*open*_) than to extending it (*G*_*extend*_). The cost of a sequence of gaps of length *x* in the affine gap model is *γ*(*x*)=*G*_*open*_+(*x*−1)∗*G*_*extend*_.

The Gotoh algorithm calculates three DP matrices: *H*, *E* and *F*, where *H* keeps track of matches/mismatches and *E* and *F* keep track of gaps in each sequence. Time and space complexities are quadratic. Matrix *H* is calculated with Eq. 3 and matrices *E* and *F* use Eqs. 4 and 5, respectively [18]. The optimal score is the value of cell *H*_*m*+1,*n*+1_. To do the traceback, the arrows are followed in matrix *H* when a match/mismatch occurs or in matrices *E* and *F*, to track multiple gaps [18].

3 $$ H_{i,j}=\max \left\{\begin{array}{l} 0 \\ E_{i,j} \\ F_{i,j} \\ H_{i-1,j-1} - p(i,j) \\ \end{array}\right.  $$

4 $$ E_{i,j}=\max \left\{\begin{array}{l} E_{i,j-1}-G_{ext} \\ H_{i,j-1}-G_{first} \\ \end{array}\right.  $$

5 $$ F_{i,j}=\max \left\{\begin{array}{l} F_{i-1,j}-G_{ext} \\ H_{i-1,j}-G_{first} \\ \end{array}\right.  $$

#### Linear space

The quadratic space complexity of NW, SW and Gotoh imposes severe restrictions when long sequences are compared. In such cases, linear space algorithms must be used. Hirschberg [19] proposed one of the first linear space algorithms for exact pairwise global sequence comparison [19]. The algorithm employs a recursive divide and conquer strategy that works as follows. First, the DP matrix is computed in linear space from the beginning to the middle row (*i*∗), storing only the last row calculated. Second, the DP matrix is calculated from the end to the middle row over the reverses of the sequences. The algorithm then uses these two middle rows and obtains the position where the addition of both columns *j* is maximal. This position is called midpoint and it corresponds to an element that belongs to the optimal alignment [19]. The midpoint divides the matrix into two smaller submatrix, which are processed recursively, until trivial solutions are found.

Myers and Miller [20] (MM) adapted Hirschberg’s algorithm to the affine gap model (“Affine-gap” section), using two additional vectors to treat situations where a sequence of gaps occurs. Let $i* = \frac {m}{2}$ be the middle row of the DP matrices, *CC*(*j*) be the minimum cost of a conversion of *S*_0_[1..*i*∗] to *S*_1_[1..*j*], *DD*(*j*) be the minimum cost of a conversion of *S*_0_[1..*i*∗] to *S*_1_[1..*j*] that ends with a gap, *RR*(*n*−*j*) be the minimum cost of a conversion of *S*_0_[*i*∗..*m*] to *S*_1_[*j*..*n*] and *SS*(*n*−*j*) be the minimum cost of a conversion of *S*_0_[*i*∗..*m*] to *S*_1_[*j*..*n*] that begins with a gap.

To find the midpoint of the alignment, the algorithm realizes a *matching procedure* against a) vectors *CC* with *RR* and b) vectors *DD* with *SS*. The midpoint is the coordinate (*i*∗,*j*∗), where *j*∗ is the position that satisfies the maximum value in Eq. 6.

6 $$ {max}_{j \in [0..n]} \left\{ max \left\{ \begin{array}{l} CC(j)+RR(n-j)\\ DD(j)+SS(n-j) - G_{open}\\ \end{array} \right. \right.   $$

As in Hirshbergś, after the midpoint is found, the matrix is recursively split into smaller submatrix, until trivial solutions are found.

### Heuristic algorithms

Usually, a given protein sequence is compared against thousands or even millions of sequences that compose genomic databases. Also, two long DNA sequences with more than a million base pairs are often compared.

In these scenarios, the use of exact algorithms such as NW and SW is often prohibitive in terms of execution time. For this reason, faster heuristic methods for local alignment were proposed which do not guarantee that the optimal result will be produced. Usually, these heuristic methods are evaluated using the concepts of sensitivity and sensibility. Sensitivity is the ability to recognize as many significant alignments as possible, including distantly related sequences. Selectivity is the ability to narrow the search in order to discard false positives [17]. Typically, there is a tradeoff between sensitivity and sensibility.

The FASTA (FAST-All) algorithm [21] was proposed in 1988 and it computes local alignments of DNA or protein sequences. It is based on FastP [22], which is a heuristic algorithm to compare a protein sequence to a genomic database composed of several protein sequences.

BLAST (Basic Local Alignment Search Tool) [23] was proposed in 1990 and it is based on FASTA. Nowadays, it is the most widely used heuristic tool for local sequence alignment. Like FASTA, the BLAST algorithm assumes that significant alignments have words of length *w* in common and it is divided into three well-defined phases: seeding, extension and evaluation.

The original BLAST algorithm searched for local alignments without considering gaps. In 1996 and 1997, two improved gapped versions of the original BLAST, NCBI-BLAST2 [24] and WU-BLAST2 [25], were proposed.

## Related work

The Smith-Waterman (SW) algorithm has become very popular over the last decade to calculate the optimal pairwise comparison of (1) two DNA/RNA sequences or (2) a protein sequence (query) to a genomic database which is composed of several sequences.

Both scenarios have been parallelized in the literature [26, 27], but fine-grained parallelism applies better to the first scenario due to the amount of data and computation involved, and therefore fits better into many-core platforms. Among them, we find Intel Xeon Phis [28], Nvidia GPUs using CUDA [29], and even multi-GPU using CUDAlign 4.0 [30], which is our departure point to analyze cost, performance and power efficiency along this work.

Studies that deal with energy consumption are becoming relevant in DNA sequence comparison applications, which motivates to provide methodologies to measure energy in this context.

Cheah et al. [31] apply an application specific integrated circuit (ASIC) design flow to decrease the power consumption of an accelerator that compares biological sequences. Reduced clock cycles and dynamic frequency scaling are employed to minimize the energy cost.

Hasan and Zafar [32] present a thorough performance versus power consumption study for bioinformatics sequence alignment using distinct field programmable gate arrays (FPGAs). A linear systolic array was used to implement the SW algorithm in those FPGA platforms.

Zou et al. [33] analyze performance and power for SW on CPU, GPU and FPGA, declaring the FPGA as the overall winner. Nevertheless, the authors do not measure real-time power dynamically, but simplify the measurements with a static value for the whole run. Moreover, they use models from the first and second Nvidia GPU generations (GTX 280 and 470), which are, by far, the most inefficient CUDA families as far as energy consumption is concerned (just 4-6 GFLOPS/W versus 15-17 GFLOPS/W in the third generation and up to 40 GFLOPS/W in the fourth generation).

Benkrid et al. [27] perform a similar analysis on CPU, FPGA and GPU, even including the Cell BE. They report 0.085 GCUPS for the CPU, 1.2 GCUPS for the GPU, 3.84 GCUPS for Cell BE and 19.4 GCUPS for the FPGA. Eight years later, we attain 276.53 GCUPS on a Pascal GPU and 37.67 GCUPS on a Altera Stratix V FPGA as we will show later on “Comparison with other devices and implementations” section, where we compare our work with theirs. Additionally, we measure real power on physical wires at real-time, instead of using static values or even TDPs like contemporary authors do [34].

## CUDAlign implementation on GPUs

SW executes in two phases: (1) compute the DP matrix and (2) traceback. Most of the time is spent in phase 1 and, therefore, this phase is often parallelized. Equation 2 exposes the data dependency among the DP matrix cells, i.e., *H*_*i*,*j*_ depends on three other cells: *H*_*i*−1,*j*_, *H*_*i*−1,*j*−1_ and *H*_*i*,*j*−1_. The parallelization of this kind of dependency is traditionally made by the wavefront method [35], in which each diagonal of the DP matrix is computed in parallel.

**Fig. 4:**
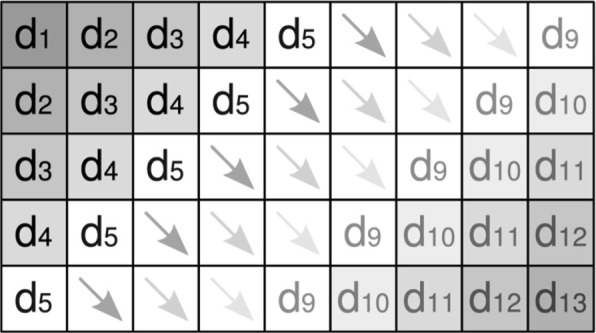
The wavefront method

The wavefront method is illustrated in Fig. 4. At the beginning (step 1) a single cell is calculated in diagonal *d*_1_. In step 2, both cells of diagonal *d*_2_ are computed in parallel. In steps 3 to 5, the number of cells that can be calculated in parallel increases until it reaches the maximum parallelism in diagonal *d*_5_. The maximum parallelism is kept in the computation of diagonals *d*_5_, *d*_6_, *d*_7_, *d*_8_ and *d*_9_ (five cells are calculated in parallel). In the computation of diagonals *d*_10_ to *d*_12_, the parallelism decreases until a single cell is computed in diagonal *d*_13_. In the wavefront method, parallelism is non-uniform and it is explored in a limited way when the wavefront is being filled (beginning of the computation) and when it is being emptied (end of the computation).

CUDAlign [29] obtains the alignment of long sequences with variants of SW and Myers-Miller (see stages and phases summarized in Table 1). The GPU calculates a single SW matrix using all many-cores in a fine grained way, and data dependencies force neighbour cores to communicate in order to exchange border elements.

**Table 1:** Summary of CUDAlign stages, including the SW phase it belongs to and the processor where it is executed

Stage	Description	SW Phase	Who
1	Obtaining the optimal score.	1	GPU
2	Partial traceback.	2	GPU
3	Splitting partitions.	2	GPU
4	Balanced splitting.	2	CPU
5	Concatenating the optimal alignment.	2	CPU
6	Alignment visualization (optional).	2	CPU

**Fig. 5:**
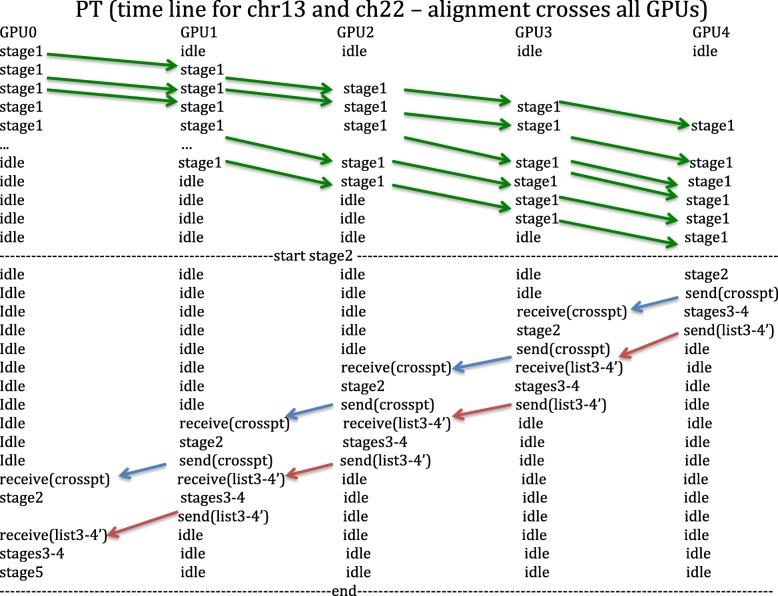
Timeline for a multi-GPU sequence alignment (4 GPUs)

When CUDAlign is executed in a platform composed of multiple GPUs, a challenging scenario arrises. The computation of the SW matrix is split among the GPUs and each GPU calculates a subset of columns. GPUs are arranged logically in a linear way, sending the border column elements to the next GPU. Communication between neighbor GPUs is overlapped with computations, i.e., it is carried by asynchronous CPU threads while the GPUs keep computing (Fig. 5).

CUDAlign 4.0 [30] introduces two strategies to optimize the traceback phase in multi-GPU executions: Pipeline Traceback (PT) and Incremental Speculative Traceback (IST). PT executes in parallel Stages 2 to 4 from different partitions. First, Stage 1 is executed, and then starts Stage 2 in last GPU. When last GPU gets the crosspoint, this is sent to previous GPU, which starts the execution of Stage 2 for the neighboring partition. The executions of Stages 2, 3 and 4 might be calculated in pipeline at each GPU.

The IST strategy for stage 2 was built based on PT strategy. IST uses the knowledge obtained in several analyses, particularly that the best score in border columns tend to coincide with the crosspoints in them. With this in mind, IST speculates crosspoints in intermediate border columns and Stage 2 in Fig. 5 can be executed in parallel before the optimal alignment crosspoints are known.

### CUDAlign versions

CUDAlign was implemented in CUDA, C++ and pthreads. Results obtained in a large GPU cluster using long DNA sequences present good scalability for up to 16 GPUs [30]. Using CUDAlign 4.0 and input data set described in “Experimental setup” section, a 14.8x speedup was attained. In this case, the execution time was reduced from 33 h and 20 min (single GPU) to 2 h and 13 min (16 GPUs).

Table 2 presents the set of improvements and optimizations performed on CUDAlign over the years. Along this work, we use CUDAlign 4.0.

**Table 2:** Summary of CUDAlign versions

Version	Major contributions	Ref.
1.0	Uses SW with affine gap (Gotoh) to compare long sequences on GPUs. It ouputs the optimal score and the end coordinates of the optimal alignment.	[50]
2.0	Includes an adapted version of Myers-Miller to retrieve the optimal alignment in linear space.	[51]
2.1	Block pruning optimization.	[29]
3.0	Multi-GPU version for SW phase 1 (distributes the matrix, overlaps computations with communications).	[49]
4.0	Multi-GPU version for SW phases 1 and 2, including Incremental Speculative Traceback (IST) to accelerate the multi-GPU retrieval of the optimal alignment.	[30]
MASA	Multi-platform architecture for sequence aligner, enabling versions to run on (1) multicore CPU using OpenMP, (2) multicore CPU using OmpsSs, (3) manycore GPU using CUDA, and (4) Xeon Phi using OpenMP.	[52]

## Experimental setup

We have conducted an experimental study on a computer endowed with an Intel Xeon server, where we have plugged different number and models of Nvidia GPUs (they will be changing depending of the experiment performed). See Table 3 for a summary of hardware features.

**Table 3:** Characterization of the infrastructure (CPU and GPUs)

Processor	Intel Xeon CPU	Nvidia GeForce GPUs			
Model	E5-2620 v4	GTX 680	GTX 980	Titan X	Titan X
Generation	Broadwell-EP	Kepler	Maxwell	Maxwell	Pascal
Year	2017	2013	2015	2016	2017
Number of cores	8	1536	2048	3072	3584
Core speed (MHz)	2100	1006	1216	1000	1405
GFLOPS (peak)	16,8	3090	4980	7144	10157
Memory Size (GB)	64	2	4	12	12
” Speed (MHz)	2400	6000	7000	7000	10000
” Width (bits)	256	256	256	384	384
” Bandwidth (GB/s)	76,8	192	224	336	480

As input, we used real DNA sequences obtained from the National Center for Biotechnology (NCBI) [36]. We compare homologous chromosomes from human and chimpanzee genomes, as it has been observed high similarity in evolutionary studies on the human species [37], in particular for chromosomes 16 [38], 22 [39] and Y [40]. Our selection is summarized in Table 4, where comparisons are named chr22, chr21, 47M, chrY, following names found in [29, 30]. DNA sequences from all those chromosomes are compared in the results presented in Table 5. From Table 6 on, we always use as input the pair of sequences from the chromosome 22 comparison (chr22) between the human (51.30 MBP [41], accession number NC_000022.11) and the chimpanzee (49.73 MBP [42], accession number NC_006489.4).

**Table 4:** The input data set used along our experiments

DNA sequence comparison	chr22	chr21	47M	chrY
Human size	51.304.566	48.129.895	46.944.323	59.373.566
Human accession #	NC_000022.11	NC_000021.8	NC_000021.7	NC_000024.9
Chimpanzee size	49.737.984	46.489.110	32.799.110	26.342.871
Chimpanzee accession #	NC_006489.4	NC_006488.2	BA000046.3	NC_006492.3
Petacells	2.55	2.24	1.54	1.56
Score	31.510.791	36.006.054	27.206.434	1.394.673
Length	51.929.087	48.579.349	33.583.457	2.283.191
Coverage	98.9%	99.0%	70.5%	6.0%
Matches	88.5%	91.9%	94.4%	88.1%
Mismatches	3.8%	1.1%	1.5%	2.0%
Gaps	7.7%	7.1%	4.1%	10.0%

**Table 5:** Power, execution times and energy consumption on four GTX980 GPUs for different comparisons

Comparison	Stage 1	Stage 2	Stage 3	Total	
	Average power (watts per GPU)				GCUPS/W
chr22	101.11 W	116.26 W	77.27 W	101.33	0.55
chr21	102.11 W	116.47 W	78.89 W	102.18	0.56
47M	104.37 W	117.12 W	76.33 W	104.50	0.54
chrY	103.25 W	119.63 W	77.00 W	103.26	0.56
	Execution time (seconds)				GCUPS
chr22	11161.92 s	185.20 s	14.25 s	11361.38 s	224.60
chr21	9687.36 s	61.49 s	11.03 s	9759.89 s	229.25
47M	6694.95 s	88.25 s	9.05 s	6792.26 s	226.68
chrY	6798.12 s	3.99 s	0.07 s	6802.18 s	229.93
	Energy consumption (kilojoules per GPU)				Cost
chr22	1128.63 kJ	21.53 kJ	1.10 kJ	4x 1151.27 kJ	0.1660 €
chr21	989.26 kJ	7.16 kJ	0.87 kJ	4x 997.29 kJ	0.1440 €
47M	698.82 kJ	10.34 kJ	0.69 kJ	4x 709.85 kJ	0.1024 €
chrY	701.94 kJ	0.48 kJ	0.00 kJ	4x 702.42 kJ	0.1012 €

We also use GCUPS (GigaCells Updated Per Second) as performance metric, GCUPS/W as power efficiency metric and overall costs in euros (shown for all GPUs involved and on an average fare of 0.13 €/kWh)

**Table 6:** Power, execution times and energy consumption on different number of GTX980 GPUs for the chr22 comparison

No. GPUs	Stage 1	Stage 2	Stage 3	Total	Versus 2 GPUs	
	Average power (watts)					GCUPS/W
4	101.11 W	116.26 W	77.27 W	101.33 W		0.55
3	101.53 W	108.16 W	78.79 W	101.62 W		0.59
2	100.30 W	114.68 W	76.74 W	100.38 W		0.57
	Execution time (seconds)					GCUPS
4	11161.92 s	185.20 s	14.25 s	11361.38 s	1.96x	224.59
3	14719.32 s	253.72 s	17.70 s	14990.76 s	1.49x	170.21
2	22080.04 s	159.77 s	23.17 s	22262.99 s		114.62
	Energy consumption (kilojoules)					Cost
4	1128.63 kJ	21.53 kJ	1.10 kJ	4x 1151.27 kJ	1.03x	0.1660 €
3	1494.60 kJ	27.45 kJ	1.40 kJ	3x 1523.44 kJ	1.02x	0.1650 €
2	2214.77 kJ	18.32 kJ	1.78 kJ	2x 2234.88 kJ		0.1614 €

We also compare performance and power consumption versus the execution on 2 GPUs

The execution of the required stages on a multi-GPU platform required two modifications in CUDAlign 4.0: (1) partitioning the input sequences to fit them in texture memory, and (2) saving extra rows to the file system as marks to be used in later stages to find crosspoints with the optimal alignment. Furthermore, our experimental analysis is done on the first three stages of CUDAlign, which are the ones executed on GPUs as shown in Table 1. Stages 4 and 5 contribute with marginal execution times, and porting codes to the GPU would not be amortized, whereas stage 6 represents the external visualization.

## Monitoring energy

**Fig. 6:**
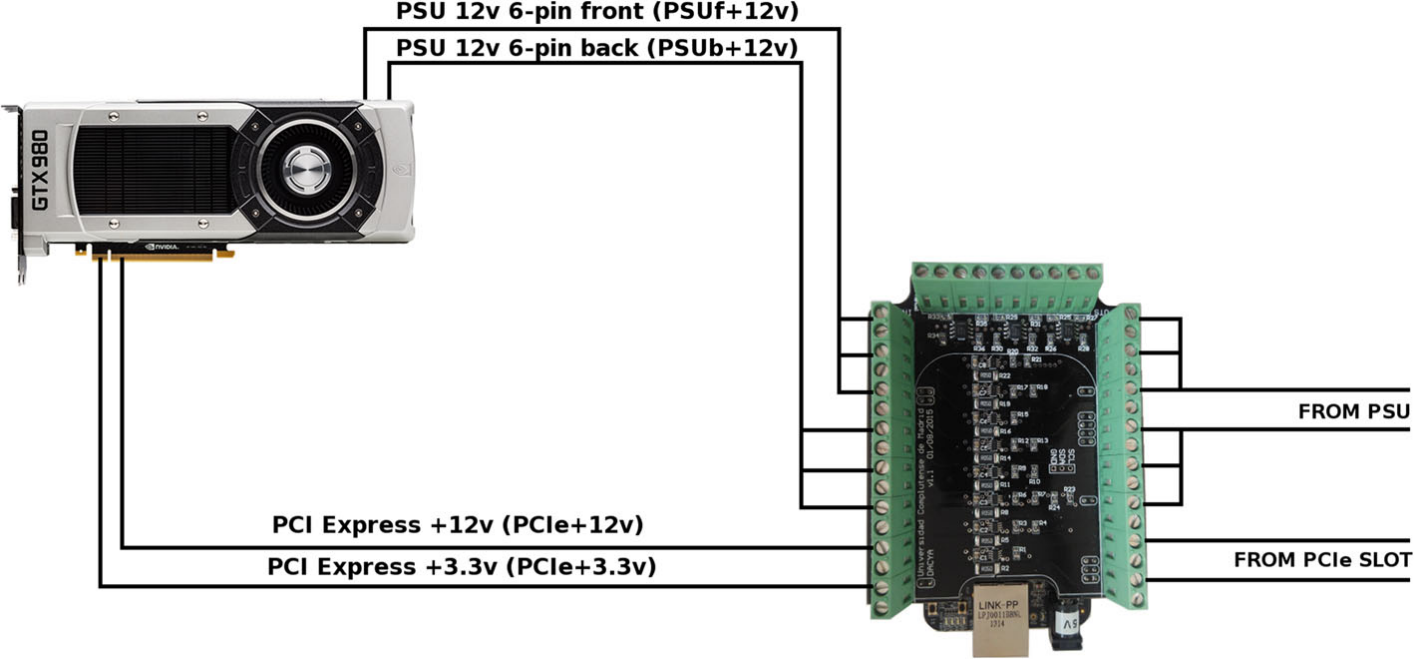
Infrastructure for measuring energy on GPUs. Wires, slots, cables and connectors for measuring energy on GPUs

Our system to measure current, voltage and wattage was built based on a Beaglebone Black, an open-source hardware [43] combined with the Accelpower module [44], which has eight INA219 sensors [45]. As described in [46], we consider two power pins on the PCI-express slot (12 and 3.3 volts) plus six external 12 volts pins coming from the power supply unit in the form of two supplementary 6-pin connectors (see Fig. 6).

The Accelpower module uses a modified version of pmlib library [47], a software package specifically created for monitoring energy. A server daemon collects power data from devices and sends them to the clients, together with a client library for communication and synchronization with the server.

**Fig. 7:**
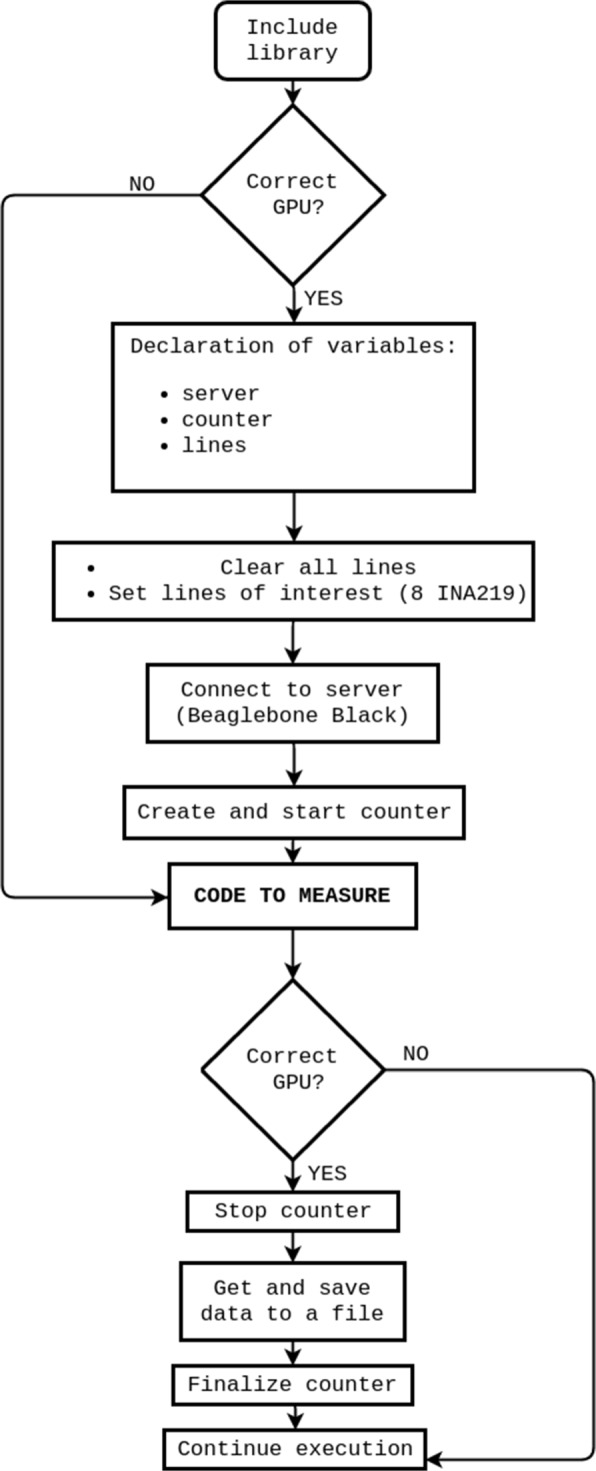
Flow chart for measuring energy on a code excerpt when running on the GPU

Our procedure for measuring energy begins with a start-up of the server daemon. Then, the CUDAlign 4.0 source code was modified in order to include the measurement calls within the GPU code as shown in Fig. 7. Before launching the code, we have to (1) declare pmlib variables, (2) clear and set the wires which are plugged to the server, (3) create a counter and (4) start it. At the end of the GPU execution, we (5) stop the counter, (6) get the data, (7) save them to a.csv file, and (8) finalize the counter.

## Results

We study performance and power efficiency on GPUs from different perspectives, studying the influence of a wide variety of issues, namely:

1. The input data volume.
2. The computational stage within SW.
3. The multi-GPU factor.
4. The cost of speculative executions.
5. Comparison among GPUs (different generations and models).
6. Comparison with other accelerators (Xeon Phis) and low-power devices (FPGAs).

Now we dedicate a different subsection to analyze each of them separately.

### Influence of the input data set

We have executed the modified CUDAlign 4.0 version using four different comparisons (see Table 4) on a multi-GPU environment composed of four GeForce GTX 980 GPUs. Table 5 includes the numbers coming from this first experiment. Wattage is slightly sensitive to workload, around 3% higher on smaller comparisons (47M and chrY). Execution time is proportional to the number of Petacells computed on each comparison, and finally, energy consumption and costs follow this tendency too. Performance keeps stable around 225-230 GCUPS (Giga-Cell Updates per Second), and power efficiency remains constant in 0.55 GCUPS/W, which is not a great value, but the use of four GPUs here penalizes it. We will see higher efficiency on a single GPU later on.

Overall, we expect the compared sequences to play a more decisive role on smaller data volumes, say Mega-Cells or even Giga-Cells. But when exceeding the Peta-Cells threshold, the GPU already reaches a stationary behaviour that stabilizes power over time regardless of input/output transitions, and it is logical to find solid performance and power efficiency. Since the influence of the compared sequences is negligible, we will continue our analysis just using chr22 in remaining executions.

### Behaviour of every computational stage

Table 5 also shows that stage 1 predominates for the execution time of SW, and that wattage keeps stable around 100 watts in that stage for all sequences. From that point, power goes up around 15% for stage 2, and goes down around 25% for stage 3. We find an explanation for this if we look at the energy budget for Kepler and Maxwell GPUs (see Table 7): Fetching operands costs more than computing on them. Therefore, stage 1, which is the most computationally intensive, keeps on an intermediate point. In stage 2, where communications are more often, average power increases. And finally, we have stage 3, almost negligible in elapsed time, but performing selective operations with texture memory and disk as we already mentioned at the end of “Experimental setup” section. File operations are offloaded to a different subsystem of the computer, and they escape to our measurement system, so the GPU is mostly idle during that time, which reduces average power and compensates those expensive DRAM operations through a much larger time window.

**Table 7:** Energy budget on a 28 nm. manufacturing process chip (all Kepler and Maxwell GPUs)

	Computational task performed on the GPU	Power consumption (energy in picojoules)
Computation	Add operator using integer operands (ALU)	0.4
	Mul operator using fp64 operands (FPU)	25
	Fused multiply-add on fp64 operands (FPU)	40
Data movement	Transition (milimeter traversed per bit)	0.2
	On-chip fp64 communication [1, 10, 20 mm.]	[3, 64, 250]
	Efficient off-chip link	500
Memory access	Local access to a register file	2
	256-bit access to on-chip 8 KB. SRAM cache	50
	DRAM read/write (for an entire cache line)	16000

**Fig. 8:**
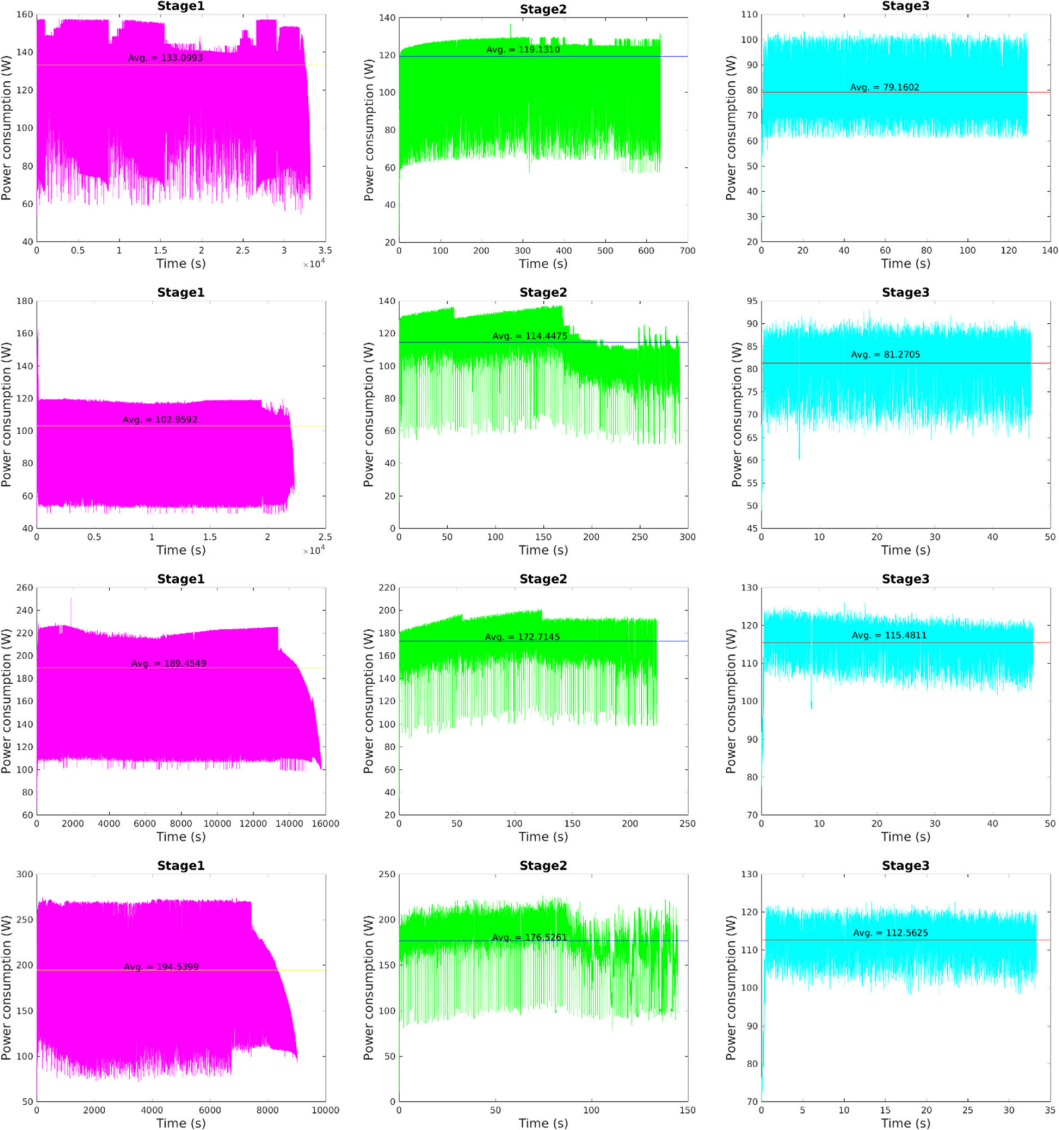
Power consumption running the chr22 comparison on a single GPU for different SW stages and GPU models (first row for GTX680, second row for GTX980, third row for Titan Maxwell and last row for Titan Pascal

Table 8 summarizes gains (in time reduction) and losses (as extra energy costs) on all scenarios of our multi-GPU execution for the chr22 sequence comparison, comparing 3 and 4 GPUs taking as baseline the first multi-GPU execution (2 GPUs). Stage 1 presented scalable results with a time reduction of almost 50% when doubling the number of GPUs from 2 to 4, at the expense of less than 2% in the overall energy budget. Stage 2 slowdowns the execution time due the insufficient level of parallelism when using few GPUs, what causes a serial execution of the Stage 2 among GPUs. Since Stage 3 executes in parallel on each GPU, speedups increase up to 38.5%. Finally, we have a solid conclusion on four GPUs, with time being reduced 50% at the expense of doubling the energy budget. Being Stage 1 the one with highest workload, the total execution time and energy usage follows its behaviour. Figure 8 provides details about the dynamic behaviour over time for each of the SW stages on different GPU models.

**Table 8:** Savings (in execution time) and penalties (in energy cost) when accelerating SW for the chr22 comparison on 4 and 3 GPUs taking as baseline the twin GPUs execution

	Stage 1		Stage 2		Stage 3		Total	
No. GPUs	Savings (time)	Penalty (energy)	Savings (time)	Penalty (energy)	Savings (time)	Penalty (energy)	Savings (time)	Penalty (energy)
4	49.96%	1.91%	-15.92%	135.04%	38.50%	23.60%	48.97%	3.03%
3	33.34%	1.22%	-58.80%	124.75%	23.61%	17.98%	32.67%	2.25%

### Changing the number of GPUs

Table 6 shows power and execution times when running SW for the chr22 comparison on a multi-GPU environment composed of 2, 3 and 4 GTX980 GPUs. We spend more than 6 h on two GPUs, and progressively reduce this time to less than a half using 4 GPUs.

As expected, power consumed by each GPU remains stable regardless of the number of GPUs active during the parallelization process. Execution times exhibit good scalability on stage 1 and 3 and somehow unstable for stage 2. Because GPUs keep computing on stage 1 most of the time, the overall energy cost is heavily influenced by this stage. Basically, doubling from 2 to 4 GPUs cuts execution times in half and doubles performance in GCUPS, with a small reduction on power efficiency: 0.57 GCUPS/W on two GPUs versus 0.55 GCUPS/W on four GPUs.

### Energy costs for speculative executions

CUDAlign 4.0 executes the two SW algorithm phases (“Algorithm SW for local alignment” section) in six stages. In the first stage, the DP matrix is computed by multiple GPUs, which asynchronously communicate border elements to the right neighbour in order to find the optimal score. In the remaining stages, the traceback phase of SW speculates the location of the optimal alignment incrementally over the values calculated so far, thus anticipating results. Otherwise, its inherent serial execution would consume more than 50% of the overall execution time, depending on the sizes of the sequences. The speculative strategy, called IST (Incremental Speculative Traceback), has already been proven to be an effective mechanism for reducing the execution time, particularly on large-scale comparisons mapped to a wide number of GPUs [30].

To guarantee an effective reduction of the execution time, we speculate on two premises: (1) during the time the GPUs are otherwise idle, and (2) showing a very high speculation hit ratio. In terms of power consumption, the first premise consumes extra energy, which would only be amortized through a much shorter execution. And mispredictions may also jeopardize the GFLOPS/w ratio.

**Fig. 9:**
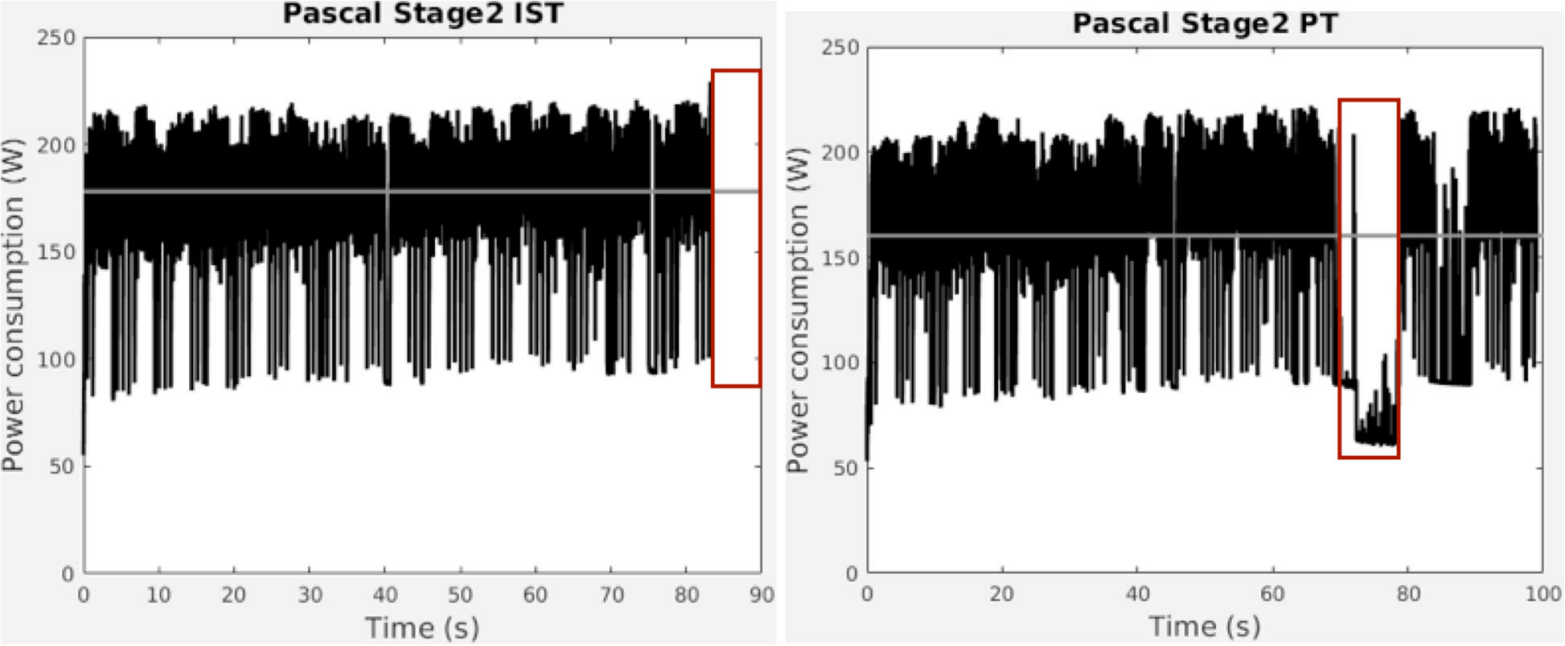
Power consumption running the chr22 comparison on 2 Pascal GPUs without (right) and with (left) speculative execution

In order to analyze energy costs and establish amortization times for CUDAlign 4.0 in multi-GPU environments, we have monitored power consumption at real-time on a dual GPU execution performed on Titan Maxwell GPUs. Figure 9 shows the dynamic behaviour of a GPU when it speculates (IST - on the left) and when it does not (PT - on the right). We see that speculation skips inactivity (boxed on the right), and the GPU ends 18% earlier. That produces average power to increase 11%, but with energy savings of 6.5% when we speculate.

Table 9 summarizes pros and cons of the speculative approach. We need at least a 2 hit/miss ratio to waive energy penalties, and when doing so, we would save 12% of execution time as starting threshold.

**Table 9:** Speculative costs on a dual GPU execution using Pascal GPUs and chr22 comparison

Execution	Time	Avg. Power	Energy
Regular (PT)	99.04 s	160.21 W	15867.19 J
Speculative (IST)	83.39 s	177.97 W	14840.91 J
Comparison	-18%	+11%	-6.5%
Spec.	Time	Avg. Power	Energy
Hit	83.39 s	177.97 W	-6.5%
Miss	99.04 s	177.97 W	+11.0%

### Comparison among GPUs

Our next analysis compares different GPU generations and models. We have GPUs coming from three different generations (one Kepler, two Maxwells and one Pascal), and from two different budgets (two mid-end GTXs and two high-end Titans). Figure 8 illustrates the dynamic behaviour of power consumption for every GPU model (represented in rows, newer are lower) and CUDAlign stage (in columns).

Going stage by stage, we can see that:

1. Stage 1 reduces power in its final part on Titan GPUs.
2. Stage 2 has a different pattern on every GPU, predominating regions of lower power on newer GPUs, particularly at the end of the process.
3. Stage 3 shows the same pattern in all cases, and average power is higher on newer GPUs, but this is caused by a much higher throughput when computing and bandwidth when communicating, leading to power savings overall.

Table 10 provides more detailed results. In general, Titan Maxwell disappoints in power efficiency but fulfills expectations in acceleration, whereas Titan Pascal is outstanding in both respects. We have built a GPU comparison taking GTX 980 (2015) as baseline (see sixth column in Table 10. Titan Maxwell (2016) improves 30% the execution time but penalizes energy in the same percentage. Titan Pascal (2017) is able to reduce time by 59% and also energy by 23%. Finally, Kepler, our 2013 model, increases execution time by 50% and almost doubles energy requirements.

**Table 10:** Summary of CUDAlign stages, including the SW phase it belongs to and the processor where it is executed

GPU model	Stage 1	Stage 2	Stage 3	Total	Versus GTX 980	
	Average power (watts)					GCUPS/W
GTX 680 (Kepler)	133.10 W	119.13 W	79.16 W	132.63 W	+28%	0.57
GTX 980 (Maxwell)	102.95 W	114.44 W	81.27 W	103.06 W		1.09
Titan X Maxwell	189.45 W	172.71 W	115.48 W	189.00 W	+83%	0.84
Titan X Pascal	194.54 W	176.52 W	112.56 W	193.96 W	+88%	1.42
	Execution time (seconds)					GCUPS
GTX 680 (Kepler)	33207.27 s	642.37 s	128.70 s	33978.34 s	+50%	75.10
GTX 980 (Maxwell)	22302.24 s	291.50 s	46.65 s	22640.40 s		112.71
Titan X Maxwell	15870.33 s	222.11 s	46.51 s	16138.95 s	- 29%	158.10
Titan X Pascal	9114.21 s	144.75 s	33.02 s	9291.98 s	- 59%	274.62
	Energy consumption (kilojoules)					Cost
GTX 680 (Kepler)	4419.88 kJ	76.52 kJ	10.18 kJ	4506.58 kJ	+93%	0.1627 €
GTX 980 (Maxwell)	2296.22 kJ	33.36 kJ	3.79 kJ	2333.37 kJ		0.0842 €
Titan X Maxwell	3006.63 kJ	38.36 kJ	5.37 kJ	3050.36 kJ	+30%	0.1101 €
Titan X Pascal	1773.07 kJ	25.55 kJ	3.71 kJ	1802.33 kJ	- 23%	0.0650 €

The comparison on sixth column takes GTX 980 as baseline

Concerning power efficiency, GTX models behave better than Titans. And we are nicely surprised by energy costs: in less than five years, we have been able to reduce the energy cost of running our algorithm by as much as 60%.

### Comparison with other devices and implementations

For Megabase DNA sequences, the SW matrix is several Petabytes long, and so, few implementations allow alignments for DNA sequences longer than 10 MBP (Million Base Pairs) like CUDAlign does.

SW# [48] performed a CUDA implementation of dynamic programming algorithms for local alignment. With an emphasis on memory optimizations, SW# was the first alternative to CUDAlign to produce sequence alignments on genome-wide scale, reporting a performance few hundred times faster than a counterpart CPU version.

More recently, Rucci et al. [34] have developed an OpenCL version of SW to study performance and power efficiency on Intel Xeon Phis and, overall, FPGAs, which has traditionally been the most power efficient devices for SW [27, 33]. Using an Altera Stratix V FPGA, they analyze the influence of data types for the matrix elements, showing that performance can be almost doubled when migrating from long int (32 bits) to int (16 bits), and from here to char (8 bits). Unfortunately, those smaller data types overflow when using their scoring function on large sequences, so they conclude that FPGAs are faster than GPUs on small sequences (up to 200K x 200K matrices), and achieve the best GCUPS/W ratios.

Table 11 summarizes results for those counterpart implementations and also from previous CUDAlign versions. Running on the same device (the GTX 980 GPU), our code improves 1.39x performance and 2.25x power efficiency those results reported in [49]. And using the Pascal GPU, we are able to increase performance and power efficiency an additional 2.45x and 1.28x factors, respectively. Note that those results in Table 11 coming from other sources do not measure real power consumption like we do, but estimate it using the TDP reported by the manufacturer.

**Table 11:** Summary of SW implementations on accelerators and low-power devices over the past five years

Device	Hardware model	Power	Implementation	Input size	GCUPS	GCUPS/W	Ref.
FPGA	Altera Stratix V	25 W ^(a)^	OpenCL	23Mx25M	37.67	1.50	[34]
Accel.	Intel Xeon Phi 3120P	270 W ^(a)^	OpenCL	23Mx25M	30.36	0.12	[34]
GPU	Nvidia Tesla K20	225 W ^(a)^	SW#	23Mx25M	44.19	0.19	[48]
GPU	” Tesla K20	225 W ^(a)^	CUDAlign 3.0	23Mx25M	40.69	0.18	[49]
GPU	” GeForce GTX 980	165 W ^(a)^	SW#	23Mx25M	67.55	0.41	[48]
GPU	” GeForce GTX 980	165 W ^(a)^	CUDAlign 3.0	23Mx25M	84.84	0.51	[49]
GPU	” GeForce GTX 980	103.06 W	CUDAlign 4.0	51Mx50M	112.71	1.09	
GPU	” Titan X Maxwell	189.00 W	CUDAlign 4.0	51Mx50M	158.10	0.84	
GPU	” Titan X Pascal	193.96 W	CUDAlign 4.0	51Mx50M	276.53	1.43	

^a^Authors do not measure real power consumption, but estimate it using TDP (Thermal Design Power)

To dedicate few words to CPUs, Korpar et al. [48] already demonstrated that the CUDA version of SW running on GPUs is few hundred times faster than a counterpart CPU version, and given the fact that GPUs have conquered the green500.org list, we assume that CPUs are not competitive on power efficiency either. Even x86 many-cores, like the Xeon Phi 3120P endowed with 57 cores show the lowest performance and power efficiency of all configurations compared in Table 11.

FPGAs, on the other hand, are much slower than GPUs on large DNA sequences, but tough competitors regarding power efficiency. In fact, we compiled in “Related work” section several results where FPGAs clearly outperformed GPUs in GCUPS/W using GeForces coming from the first CUDA generation. Eight years and four generations later, GPU technology has turned around this situation. Table 12 summarizes speedups attained by GPUs and their deficit in energy versus FPGAs. We can see how performance gaps widens and power efficiency converges whenever GPUs reach contemporary models. And the programming effort on a FPGA is remarkable: 300 days versus just 45 days on a GPU for the implementation described in [27].

**Table 12:** GPUs contribution in acceleration and energy consumed versus a 2017 FPGA implementation using OpenCL

Platform	FPGA	GTX 980	GTX 980	GTX 980	Titan Pascal
Implementation	OpenCL	SW# (CUDA)	CUDAlign 3.0	CUDAlign 4.0	CUDAlign 4.0
Year	2017	2013	2014	2016	2017
Performance	Baseline	+79.3%	+125.2%	+213.5%	+669.2%
Power efficiency	Baseline	- 72.6%	- 66.0%	- 23.3%	- 1.3%

## Discussion and conclusions

Along this paper, we have studied GPU acceleration and power consumption on a multi-GPU environment for the Smith-Waterman method to compute, via CUDAlign 4.0, the biological sequence alignment for a set of real genome scale DNA sequences coming from human and chimpanzee homologous chromosomes retrieved from the National Center for Biotechnology Information (NCBI).

We may distinguish 6 stages within CUDAlign 4.0, and the first half have been implemented in CUDA for its acceleration on GPUs. On a stage by stage analysis, the first one is more demanding and takes the bulk of the computational time. On the other hand, power consumption was kept more stable across executions of different alignment sequences, though it suffered deviations of up to 30% across different stages.

One of the major innovations in CUDAlign 4.0 was the Incremental Speculative Traceback (IST) [30] introduced within stage 2 to estimate the point where optimal alignment crosses border columns among multiple GPUs. This strategy allows GPUs to anticipate their activation, minimizing idle times required to solve dependencies when parallelizing. Mispredictions barely affect the execution time, but they may compromise power efficiency. On a dual GPU execution performed on Titan Maxwell GPUs, we find that a 2 hit/miss ratio is required to waive energy penalties, and in that case, we will also save 12% of the execution time.

In a multi-GPU environment composed of 4 GTX980 GPUs, we reduce the computational time by half when compared to a dual GPU execution, at the expense of just 3% penalty in power usage. That way, our experiments demonstrated an efficient correlation between acceleration and extra energy required.

Overall, we have reduced execution times from 9.5 h on a Kepler GPU to just 2.5 h on Titan Pascal, with energy costs cut by 60%. Compared to FPGAs, which have an excellent reputation as low-power devices, GPUs are competitive and keep similar GFLOPS/w ratios in 2017 while maintaining the leadership as HPC accelerators for a five times faster execution.

We expect GPUs to increase their role as high performance and low power devices for biomedical applications in future GPU generations, particularly after the introduction in early 2017 of the 3D memory within Pascal models.

## Abbreviations

ASIC: Application specific integrated circuit
BLAST: Basic Local Alignment Search Tool
BLOSUM: Blocks Substitution Matrix
CUDA: Compute unified device arquitecture
DP: Dynamic programming
FPGA: Field programmable gate array
GCUPS: Giga-Cell Updates Per Second
GPGPU: General-purpose graphics processing unit
GPU: Graphics processing unit
HPC: High performance computing
IST: Incremental Speculative Traceback
MBP: Millions of Base Pairs
MM: Myers and Miller
NCBI: National Center for Biotechnology
NW: Needleman-Wunsch
PAM: Percent Accepted Mutations
PT: Pipeline Traceback
SW: Smith-Waterman
